# Eggs – a scoping review for Nordic Nutrition Recommendations 2023

**DOI:** 10.29219/fnr.v68.10507

**Published:** 2024-02-06

**Authors:** Jyrki K. Virtanen, Susanna C. Larsson

**Affiliations:** 1 School of Medicine, Institute of Public Health and Clinical Nutrition, University of Eastern Finland, Kuopio, Finland; 2 Unit of Medical Epidemiology, Department of Surgical Sciences, Uppsala University, Uppsala, Sweden; 3 Unit of Cardiovascular and Nutritional Epidemiology, Institute of Environmental Medicine, Karolinska Institutet, Stockholm, Sweden

**Keywords:** eggs, cholesterol, choline, cardiovascular disease, dietary recommendations

## Abstract

Cardiovascular diseases (CVD), type 2 diabetes (T2D), and cancer are a significant public health burden in the Nordic and Baltic countries. High intake of eggs, mainly due to its high cholesterol content, has been suggested to have adverse health effects. The purpose of this scoping review is to describe the evidence related to the impact of egg intake on health. A literature search identified 38 systematic reviews and meta-analyses on egg consumption in relation to health outcomes published between 2011 and 30 April 2022. Overall, current evidence from systematic reviews of randomized clinical trials indicates that higher egg intake may increase serum total cholesterol concentration and the ratio of low-density lipoprotein to high-density lipoprotein cholesterol, but with substantial heterogeneity in the response. However, recent evidence from observational studies does not provide strong support for a detrimental role of moderate egg consumption (up to one egg/day) on the risk of CVD, especially in the European studies. The overall evidence from observational studies indicates that egg consumption is not associated with increased risk of mortality or T2D in European study populations. There is also little support for a role of egg consumption in cancer development, although a weak association with higher risk of certain cancers has been found in some studies, mainly case–control studies. Again, no associations with cancer risk have been observed in European studies. Systematic reviews and meta-analyses of egg consumption in relation to other health-related outcomes are scarce. There are also limited data available on the associations between the consumption of more than one egg/day and risk of diseases. Based on the available evidence, one egg/day is unlikely to adversely affect overall disease risk.

## Popular scientific summary

- Eggs are nutrient-dense, a good source of high-quality protein and vitamin B_12_, and contain several other bioactive compounds.
- The health impact of egg consumption has been a controversial topic due to the content of both beneficial and unfavorable components, such as cholesterol.
- Current evidence indicates that the intake of eggs is not associated with the risk of mortality in European populations.
- Limited evidence suggests that the intake of up to one egg/day is not associated with increased risk of cardiovascular disease.
- The evidence for an association between egg intake and risk of cancer and type 2 diabetes in European populations is limited, although a modestly elevated risk of certain cancers cannot be ruled out.
- There are little data on health effects of intakes above one egg/day.

Egg is a common food-item in the diets of the Nordic and Baltic countries and an ingredient in many dishes and recipes. However, the health impact of egg consumption may be one of the most controversial issues in nutrition because, on the one hand, egg yolk is a major contributor to dietary cholesterol intake. One medium-sized egg contains approximately 200 mg of cholesterol. The average total cholesterol intake in the Nordic countries is 230–400 mg/day and 250–340 mg/day in the Baltic countries (1). The Nordic Nutrition Recommendations (NNR) has not set an upper intake level for dietary cholesterol. In the US, The National Academies recommends that dietary cholesterol intake should be as low as possible without compromising the nutritional adequacy of the diet (2). Egg also has a high content of choline that can be metabolized into trimethylamine by gut microbiota and then further converted in the liver to trimethylamine-*N*-oxide (TMAO), a purported risk factor for cardiovascular diseases (CVD) (3).

On the other hand, egg is a very nutrient dense food-item, as one medium-sized egg contains only about 75 kcal. Egg is a source of high-quality protein, all essential vitamins except vitamin C, minerals, and several bioactive compounds, and contains mainly unsaturated fat (4, 5). For example, one egg accounts for about 62% of the daily recommended intake of vitamin B_12_, 30% of selenium, 16% of iodine, and 12% of vitamin D (fineli.fi). Therefore, the health effects of egg intake are difficult to determine by considering only its high content of cholesterol or choline. Cholesterol and many of the other nutrients are present only in the egg yolk. Egg white contains mainly protein.

A number of epidemiological studies have examined the association between egg intake and the risk of various diseases, particularly CVD, type 2 diabetes (T2D), and cancer, but the results are inconclusive. The aim of this scoping review is to describe the totality of evidence for the role of egg intake for health-related outcomes as a basis for setting and updating the food-based dietary guidelines in NNR 2023 (Box 1). A literature search was conducted in PubMed to find systematic reviews and meta-analyses that had investigated the associations of egg intake with health outcomes.

Box 1 Background papers for Nordic Nutrition Recommendations 2023

- This paper is one of many scoping reviews commissioned as part of the Nordic Nutrition Recommendations 2023 (NNR2023) project (6)
- The papers are included in the extended NNR2023 report, but, for transparency, these scoping reviews are also published in Food & Nutrition Research
- The scoping reviews have been peer reviewed by independent experts in the research field according to the standard procedures of the journal
- The scoping reviews have also been subjected to public consultations (see report to be published by the NNR2023 project)
- The NNR2023 committee has served as the editorial board
- While these papers are a main fundament, the NNR2023 committee has the sole responsibility for setting dietary reference values in the NNR2023 project

## Methods

This review follows the protocol developed within the NNR2023 project (6). The sources of evidence used in this chapter follow the eligibility criteria described previously (7).

There were no *de novo* systematic reviews or qualified systematic reviews available, as defined by the NNR2023 Committee, as a source of evidence for the egg scoping review. A literature search was conducted in PubMed (last update on 30 April 2022) using the query “(eggs[MeSH Terms] OR egg) AND (“2011”[Date – Publication] : “3000”[Date – Publication]) AND humans[Filter] AND (review[filter] OR systematic review[filter] OR meta-analysis[Publication Type])”. This search resulted in 2,639 hits, of which 38 articles reported results from a systematic review or meta-analysis on egg consumption in relation to the risk of diseases. The most relevant systematic reviews and meta-analyses for setting the guidelines for egg intake are described in Table 1. The most recent and comprehensive meta-analyses were chosen for inclusion in this chapter. The list of studies related to egg intake and risk of diseases that were identified with the query but not included in setting the guidelines is provided in Table 2. The quality of included studies was evaluated using a modified version of AMSTAR 2 (6). The strength of evidence was graded using the World Cancer Research Fund criteria (46). In addition, the search detected eight systematic reviews and meta-analysis that investigated the effect of increased egg intake on disease risk factors in randomized clinical trials (RCTs) (47–54).

**Table 1:** List of included studies in setting the Nordic Nutrition Recommendations 2023 for egg intake

1st author (year), (reference)	Outcome(s)	Study type (review/meta-analysis/both)	Number of included primary studies*	Design of included primary studies (case–control/nested case–control/prospective cohort)	Exposure categories	Main findings	Evidence for heterogeneity?	Evidence for publication bias?	AMSTRAR-2 rating
*Total mortality*									
Mazidi et al. (2019) (8)	Total mortality	Systematic review and meta-analysis	7	Prospective cohorts	Highest vs. lowest intake category	HR = 1.11 (95% CI 0.97–1.26)	No	No	Critically low
Mousavi et al. (2022) (9)	Total mortality	Systematic review and meta-analysis	24	Prospective cohorts	Highest vs. lowest intake category	RR = 1.02 (95% CI 0.94–1.11) In the US studies, RR = 1.18 (95% CI 1.10–1.26); in the European studies, RR = 1.03 (95% CI 0.78–1.35); in the Asian studies, RR = 0.89 (95% CI 0.80–0.99); and in an international study, RR = 0.99 (0.90–1.08)	Yes, *P* < 0.001, *I*^2^ = 95.4% Yes, *P* < 0.001, *I*^2^ = 97.7%	Yes, Egger *P* = 0.01	High
					1 egg/week increase in intake	RR = 1.02 (95% CI 1.00–1.03) In the US studies, RR = 1.02 (95% CI 0.99–1.05); in the European studies, RR = 1.06 (95% CI 0.98–1.14); in the Asian studies, RR = 0.99 (95% CI 0.96–1.02); and in an international study, RR = 1.00 (95% CI 0.99–1.01)			
*Cardiovascular diseases*									
Mazidi et al. (2019) (8)	CHD mortality, stroke mortality	Systematic review and meta-analysis	*n* = 6 for CHD mortality, *n* = 8 for stroke mortality	Prospective cohorts	Highest vs. lowest intake category	Total mortality: HR = 1.11 (95% CI 0.97–1.26), CHD mortality: HR = 1.23 (95% CI 0.88–1.77), stroke mortality: HR = 0.72 (95% CI 0.54–0.96)	No	No	Critically low
Drouin-Chartier et al. (2020) (10)	Any CVD, CHD, stroke	Systematic review and meta-analysis	*n* = 28 for any CVD, *n* = 17 for CHD, *n* = 17 for stroke	Prospective cohorts	Highest vs. lowest intake category, 1 egg/day increase in intake	Any CVD: RR = 0.99 (0.93–1.06) for high vs. low intake, RR = 0.98 (95% CI 0.93–1.03) for 1 egg/day; CHD: RR = 0.97 (95% CI 0.91–1.04) for high vs. low intake), RR = 0.96 (95% CI 0.91–1.03) for 1 egg/day; stroke: RR = 0.96 (95% CI 0.88–1.06) for high. vs low intake, RR = 0.99 (0.91–1.07) for 1 egg/day	For any CVD, yes (1 egg/day *I*^2^ = 62.3%), for stroke, yes, (1 egg/day *I*^2^ = 71.5%), for CHD, no.	No	Low
Tang et al. (2020) (11)	Stroke	Systematic review and dose-response meta-analysis	*n* = 16 for total stroke, *n* = 9 for stroke mortality	Prospective cohorts	Highest vs. lowest intake category	Total stroke: RR = 0.92 (95% CI 0.84–1.01). In dose-response analysis, 1–4 eggs/week was associated with lower risk and over 10 eggs/week with increased risk. Stroke mortality: RR = 0.84 (95% CI 0.71–1.00).	For total stroke, yes (*I*^2^ = 64.4%, *P* < 0.001). Not reported for stroke mortality.	No	Critically low
Godos et al. (2021) (12)	Total CVD, CVD incidence, CVD mortality, total CHD, CHD incidence, CHD mortality, stroke, stroke incidence, stroke mortality, HF	Dose-response meta-analysis	*n* = 14 for CVD, *n* = 16 for CHD, n = 16 for stroke, *n* = 4 for HF	Prospective cohorts	1 egg/week increase in intake	Compared to no consumption, ≤7 eggs/week associated with lower non-fatal CVD incidence; ≤3 eggs/week with lower non-fatal CHD incidence. No associations with higher intakes or with risk of fatal or non-fatal stroke or with CVD or CHD mortality. Compared to no consumption, ≥7 eggs/week associated with higher HF risk, but only in the one study conducted in the USA.	For total CVD, yes (*I*^2^ = 71%), for CVD incidence, yes (*I*^2^ = 75%), for CVD mortality, yes (*I*^2^ = 57%), for total CHD, yes (*I*^2^ = 82%), for CHD incidence, yes (*I*^2^ = 85%); for total stroke, no; for HF, no	No	Critically low
Zhao et al. (2022) (13)	Total CVD	Systematic review and meta-analysis	41	Prospective cohorts	Per 50 g/day higher intake	RR = 1.04 (95% CI 1.00–1.08). In the US studies, RR = 1.08 (95% CI 1.02–1.14); in the European studies, RR = 1.05 (95% CI 0.98–1.14); and in the Asian studies, RR = 0.96 (95% CI 0.87–1.06).	Yes, *I*^2^ = 80.1%	No	High
Mousavi et al. (2022) (9)	CVD mortality, CHD mortality, stroke mortality	Systematic review and meta-analysis	*n* = 11 for CVD mortality, *n* = 10 for CHD mortality, *n* = 9 for stroke mortality	Prospective cohorts	Highest vs. lowest intake category	CVD mortality: RR = 1.04 (95% CI 0.87–1.23). In the US studies, RR = 1.20 (95% CI 1.10–1.31); in the European studies, RR = 1.12 (95% CI 0.85–1.48); in the Asian studies, RR = 0.84 (95% CI 0.62–1.15); and in an international study, RR = 0.93 (95% CI0.80–1.07). CHD mortality: RR = 0.98 (95% CI 0.84–1.11), stroke mortality: RR = 0.81 (95% CI 0.64–1.02).	Yes, for all combined analyses, *P* < 0.001	No	High
					1 egg/week increase in intake	CVD mortality: RR = 1.00 (95% CI 0.97–1.04). In the US studies, RR = 1.04 (95% CI 1.03–1.06); in the European studies, RR = 1.04 (95% CI 0.98–1.10); in the Asian studies, RR = 0.95 (95% CI 0.89–1.02); and in an international study, RR = 1.00 (95% CI 0.96–1.03). CHD mortality: RR = 1.00 (95% CI 0.96–1.03), stroke mortality; RR = 0.96 (95% CI 0.92–0.99)			
*Hypertension*									
Zhang et al. (2018) (14)	Hypertension	Meta-analysis	3	Prospective cohorts	Highest vs. lowest intake category	Highest vs. lowest category, RR = 0.79 (95% CI 0.68–0.91)	No	No	Low
*Type 2 diabetes*									
Drouin-Chartier et al. (2020) (15)	Type 2 diabetes	Systematic review and meta-analysis	16	Prospective cohorts	1 egg/day increase in intake	For each 1 egg/day higher intake, RR = 1.07 (95% CI 0.99–1.15). In US studies, RR = 1.18 (95% CI 1.10–1.27); in European studies, RR = 0.99, 95% CI 0.85–1.15); in Asian studies, RR = 0.82 (95% CI 0.62–1.09).	Overall *I*^2^ = 69.8%, *P* < 0.001); in US studies, *I*^2^ = 51.3%; *P* = 0.03); in European studies, *I*^2^ = 73.5%, *P* > 0.001; in Asian studies, *I*^2^ = 59.1%; *P* = 0.06	Yes, Egger *P* = 0.03, Begg *P* = 0.06	Critically low
*Cancer*									
Li et al. (2013) (16)	Bladder cancer	Meta-analysis	13	4 prospective cohorts, 9 case-control studies	High vs. low intake	RR = 1.11 (95% CI 0.90–1.35). In the US studies, RR = 1.40 (95% CI 1.05–1.86); in European studies, RR = 1.01 (95% CI 0.79–1.30); in Asian studies, RR = 0.83 (95% CI 0.52–1.32). For fried egg intake, RR = 2.04 (95% CI 1.41–2.95); for boiled egg, RR = 1.25 (95% CI 0.82–1.91).	Yes (*I*^2^ = 63.3%, *P* = 0.001)	No	Critically low
Tse et al. (2014) (17)	Gastrointestinal neoplasms	Dose-response meta-analysis and systematic review	44	7 prospective cohort studies, 37 case–control studies	Lowest vs. highest categories / <3 or ≥3 eggs/week / <3, 3–5 or ≥5 eggs/week (reference category not indicated).	For intakes of <3 and ≥3 eggs/week, OR = 1.14 (95% CI 1.07–1.22) and OR = 1.25 (95% CI 1.14–1.38), respectively. For intakes of <3, 3–5, and ≥5 eggs/week, OR = 1.13 (95% CI 1.06–1.21), OR = 1.14 (95% CI 1.01–1.29), and OR = 1.19 (95% CI 1.01–1.39), respectively. In site-specific analyses, egg intake was associated mainly with risk of cancers of the stomach, colon, and colorectum. Increased risk was found only in case–control studies. No associations were found in European studies.	No	Yes, *P* < 0.001	Critically low
Keum et al. (2015) (18)	Breast cancer, ovarian cancer, prostate cancer (only prostate cancer results included, as there are more recent SRs about breast and ovarian cancers)	Dose-response meta-analysis	10 for prostate cancer	Prospective cohorts	5 eggs/week higher intake	For total prostate cancer, RR = 1.00 (95% CI 0.88–1.14); for fatal prostate cancer, RR = 1.47 (95% CI 1.01–2.14).	No	For total prostate cancer, no, for fatal prostate cancer, yes (*P* = 0.04).	Critically low
Dong et al. (2017) (19)	Non-Hodgkin lymphoma	Meta-analysis	7	1 prospective cohort study, 6 case–control studies	High vs. low intake	Overall RR = 1.15 (95% CI 0.87–1.51). RR = 1.06 (95% CI 0.88–1.27) for the prospective cohort study, RR = 1.15 (95% CI 0.82–1.62) for the case-control studies.	Yes. Overall *P* < 0.001, *I*^2^ = 85.3%, for case-control studies *P* < 0.001, *I*^2^ = 86.6%	No	Low
Luo et al. (2019) (20)	Brain cancer or glioma	Meta-analysis	5	Case–control studies	Highest vs. lowest intake category	For brain cancer, RR = 1.00 (95% CI 0.55–1.81). For studies with ≥200 cases (n = 2), RR = 1.57 (95% CI 1.27–1.93). For glioma, RR = 1.47 (95% CI 0.94–2.32)	For brain cancer, yes. *P* < 0.001, *I*^2^ = 82.6%. For glioma, no.	No	Critically low
Aminianfar et al. (2019) (21)	Upper aero-digestive tract cancers	Systematic review and meta-analysis	38	4 cohort studies, 2 nested case–control studies, 32 case–control studies	Highest vs. lowest intake category	For prospective cohort studies and nested case–control studies, OR = 0.86 (95% CI 0.71–1.04). For case–control studies, OR = 1.42 (95% CI 1.19–1.68). Increased risk was found with oropharyngeal (OR = 1.88; 95% CI 1.61–2.20), laryngeal (OR = 1.83, 95% CI 1.45–2.32), oral & pharyngeal & laryngeal (OR = 1.37, 95% CI 1.12–1.67), and esophageal cancers (OR = 1.28, 95% CI 1.10–1.48). Increased risk was found only in hospital-based case–control studies, but not in population-based case–control studies.	For case–control studies, yes (*P* < 0.001, *I*^2^ = 74.8%). For prospective cohort studies and nested case–control studies, no.	No	Low
Kazemi et al. (2021) (22)	Breast cancer	Systematic review and dose-response meta-analysis	11	Prospective cohorts	50 g/day higher intake	RR = 1.03 (95% CI 0.96–1.12)	Yes, *P* = 0.03, *I*^2^ = 48.8%.	No	High
Khodavandi et al. (2021) (23)	Ovarian cancer	Systematic review and meta-analysis	9	Prospective cohorts	High vs. lowest intake category	RR = 1.12 (95% CI 0.96–1.30)	No	Not reported	Critically low
Mousavi et al. (2022) (9)	Cancer mortality	Systematic review and meta-analysis	13	Prospective cohorts	Highest vs. lowest intake category	RR = 1.20 (95% CI 1.04–1.39) In the US studies, RR = 1.33 (95% CI 1.11–1.60); in the European studies, RR = 1.10 (95% CI 0.84–1.41); and in Asian studies, RR = 1.15 (95% CI 0.92–1.44)	Yes, *P* < 0.001, *I*^2^ = 93.6% Yes, *P* < 0.001, *I*^2^ = 91.2%	No	High
					1 egg/week increase in intake	RR = 1.04 (95% CI 1.01–1.07) In the US studies, RR = 1.07 (95% CI 1.01–1.13); in the European studies, RR = 1.03 (95% CI 0.99–1.07); and in the Asian studies, RR = 1.02 (95% CI 0.98–1.06)			
*Metabolic syndrome*									
Ding et al. (2022) (24)	Metabolic syndrome	Meta-analysis	18	Prospective cohorts (*n* = 4), cross-sectional studies (*n* = 14)	Highest vs. lowest intake category	RR = 0.92 (95% CI 0.88–0.96) Prospective cohorts: RR = 0.99 (95% CI 0.77–1.26), cross-sectional studies: RR = 0.91 (95% CI 0.88–0.95)	Yes, *P* < 0.001, *I*^2^ = 47%	No	Low

* Even if an original study presents findings from two or more study populations, it is counted as one study in a meta-analysis in this table.

CVD = cardiovascular disease; CHD = coronary heart disease; CI = confidence interval; HF = heart failure; OR = odds ratio; RR = relative risk.

**Table 2:** List of excluded studies in setting the Nordic Nutrition Recommendations 2023 for egg intake

1st author (year, reference)	Outcome(s)	Study type (review/meta-analysis/both)	Number of included primary studies	Exposure categories (reference category)	Conclusion	Reason for not including in the chapter
Xie et al. (2012) (25)	Prostate cancer	Meta-analysis	9 cohort studies, 11 case–control studies	Highest vs. lowest category	No association	More recent meta-analysis available
Fang et al. (2012) (26)	Bladder cancer	Meta-analysis	4 cohort studies, 9 case–control studies	Highest vs. lowest category	No association	More recent meta-analysis available
Rong et al. (2013) (27)	CHD, stroke	Systematic review and meta-analysis	8 cohort studies	One egg/day increase	No association	More recent meta-analyses available
Li et al. (2013) (28)	CVD, type 2 diabetes	Meta-analysis	14 studies, including 10 cohort studies, 1 case–control study, and 3 cross-sectional studies	Highest vs. lowest category	Egg intake was positively associated with risk of CVD and diabetes	More recent meta-analyses available
Shin et al. (2013) (29)	CVD, type 2 diabetes	Systematic review and meta-analysis	22 cohort studies	Highest (≥1 egg/day) vs. lowest (<1 egg/week or never) category	No association	More recent meta-analyses available
Si et al. (2014) (30)	Breast cancer	Meta-analysis	5 cohort studies, 8 case–control studies	Highest vs. lowest category	Higher egg intake was associated with a modestly increased risk of breast cancer (RR 1.04, 95% CI 1.01–1.08)	More recent meta-analysis available
Zeng et al. (2015) (31)	Ovarian cancer	Meta-analysis	6 cohort studies, 6 case–control studies	Highest vs. lowest category	Higher egg intake was associated with increased risk of ovarian cancer	More recent meta-analysis available
Djoussé et al. (2016) (32)	Type 2 diabetes	Meta-analysis	12 cohort studies	Highest vs. lowest category and dose-response analysis	No overall association between infrequent egg consumption and diabetes risk, but a suggestive and modestly elevated risk of diabetes with ≥3 eggs/week was observed in US studies.	More recent meta-analysis available
Wallin et al. (2016) (33)	Type 2 diabetes	Meta-analysis	12 cohort studies	Dose-response analysis (3 times/week increase)	Increased risk of diabetes with higher egg intake in 5 US studies, but no association in 7 non-US studies.	More recent meta-analysis available
Tamez et al. (2016) (34)	Type 2 diabetes	Meta-analysis	10 cohort studies	Dose-response analysis	Egg intake was positively associated with diabetes risk in US studies, but not in non-US studies	More recent meta-analysis available
Alexander et al. (2016) (35)	CHD, stroke	Meta-analysis	7 cohort studies on CHD and 7 cohort studies on stroke	Highest vs. lowest category (generally 1/day vs. <2/week)	Intake of up to one egg/day was associated with a lower risk of total stroke, but not associated with CHD	More recent meta-analyses available
Wu et al. (2016) (36)	Breast cancer	Meta-analysis	9 cohort studies	Highest vs. lowest category	No association	More recent meta-analysis available
Khawaja et al. (2017) (37)	Heart failure	Meta-analysis	4 cohort studies	Highest (≥1/day) vs. lowest category	Higher egg intake was associated with an increased risk of HF (RR 1.25, 95% CI 1.12–1.39)	More recent meta-analysis available
Tian et al. (2017) (38)	Type 2 diabetes	Systematic review and meta-analysis	Cohort studies	Highest vs. lowest category	No association	More recent meta-analysis available
Geiker et al. (2018) (39)	CVD, type 2 diabetes	Systematic review	Observational and interventional studies	Unclear	No association	More recent meta-analyses available
Xu et al. (2019) (40)	CVD and all-cause mortality	Meta-analysis	9 cohort studies on CHD, 9 cohort studies on stroke, and 4 cohort studies on all-cause mortality	Highest vs. lowest category	≥7 eggs/week was associated with a modest reduction in stroke risk (RR 0.91, 95% CI 0.85–0.98), but not associated with CHD (RR 0.97, 95% CI 0.90–1.05) or all-cause mortality (HR 1.09; 95% CI 0.997–1.200)	More recent meta-analyses available
Zhong et al. (2019) (41)	CVD	Meta-analysis of individual-level data from 6 cohort studies	6 US cohorts using data collected between March 25, 1985, and August 31, 2016	Dose-response analysis	Each additional 0.5 egg/day was associated with higher risk of incident CVD (RR 1.06, 95% CI 1.03–1.10) and all-cause mortality (RR 1.08, 95% CI 1.04–1.11). The associations were no longer significant after adjustment for dietary cholesterol.	Includes only US studies
Bechthold et al. (2019) (42)	CHD, stroke, heart failure	Systematic review and meta-analysis	11 cohort studies for CHD, 10 cohort studies for stroke, 4 cohort studies for HF	Highest vs. lowest category and dose-response analysis	Egg intake was positively associated with HF risk (RR 1.16, 95% CI 1.03–1.31), but not associated with CHD or stroke	More recent meta-analyses available
Marventano et al. (2020) (43)	CVD, type 2 diabetes, cancer	Umbrella review; systematic review of meta-analyses	21 articles on egg intake and health outcomes	Unclear	No association with CVD, except for evidence of a possible inverse association with stroke	Umbrella review
Mah et al. (2020) (44)	Cardiometabolic health	Umbrella review; systematic review of meta-analyses	7 qualitative reviews and 16 quantitative reviews	Unclear	No association with CVD or stroke in the general population. Increased risk of HF was noted in two meta-analyses that included the same three cohort studies. Five recent meta-analyses reported no association with diabetes in the general population, although increased risk in US-based populations only was reported.	Umbrella review
Krittanawong et al. (2021) (45)	CVD, CHD	Systematic review and meta-analysis	21 cohort studies	Highest vs. lowest category	Intake of >1 egg/day was associated with reduced risk of CHD, but not associated with risk of CVD	More comprehensive meta-analysis available

CVD = cardiovascular disease; CHD = coronary heart disease; CI = confidence interval; HF = heart failure; RR = relative risk.

## Diet intake in Nordic and Baltic countries

There is some variation in egg intake between the Nordic countries (1). The lowest reported mean egg intakes are in Iceland (men: 14 g/day and women: 10 g/day) and Sweden (14 g/day in both men and women), whereas in Finland, Norway, and Denmark, the mean intakes in men range between 24 and 28 g/day and in women between 23 and 24 g/day. In the Baltic countries, the mean egg intake is the highest in Latvia (men: 40 g/day and women: 31 g/day), with slightly lower intakes in Estonia (men: 26 g/day and women: 20 g/day) and Lithuania (men: 34 g/day and women: 23 g/day). However, the variation in egg intake in all countries is large, with large standard deviations for the mean values. It should be noted that egg is a common ingredient in recipes and dishes, but only the data from Denmark include eggs in dishes.

## Health outcomes relevant for Nordic and Baltic countries

### Cardiovascular diseases

#### Overall cardiovascular disease and coronary heart disease

A meta-analysis of 41 prospective cohort studies observed that higher egg intake was associated with a higher risk of total CVD (fatal and non-fatal events combined) (RR = 1.04, 95% CI 1.00–1.08 for each 50 g/day higher egg intake) (13). The association was found mainly in the studies conducted in the USA (RR = 1.08, 95% CI 1.02–1.14), but not in the studies conducted in Europe (RR = 1.05, 95% CI 0.98–1.14) or in Asia (RR = 0.96, 95% CI 0.87–1.06). Another meta-analysis found an inverse association between egg intake and risk of non-fatal CVD with an intake of up to 7 eggs/week (no. of studies = 9, RR = 0.94, 95% CI 0.89–0.99 when compared to no egg intake) and of non-fatal coronary heart disease (CHD) with an intake of up to 3 eggs/week (*n* = 12, RR = 0.92, 95% CI 0.86–1.00 when compared to no egg intake) (12). An earlier meta-analysis of 28 prospective cohort studies did not find statistically significant associations between the egg intake and risk of any CVD or CHD outcomes, although it did find an inverse association with CVD risk in the studies conducted in Asia, but not in Europe or the USA (10). No statistically significant associations have been observed with fatal CVD (9, 12, 13) or fatal CHD (8, 12).

In summary, the strength of evidence is regarded as *Limited – suggestive* that when compared to low or no egg intake, egg intake up to one egg/day is not associated with increased risk of CVD.

#### Stroke

Regarding the incidence of any stroke, a meta-analysis of 16 prospective cohort studies did not find an overall association with stroke risk (RR = 0.92, 95% CI 0.84–1.01 for highest vs. lowest intake category), but it did find a non-linear association with egg intake so that 50–200 g/week (1–4 eggs/week) was associated with lower risk and ≥500 g/week (≥10 eggs/week) was associated with higher risk (11). When comparing extreme categories of intake, an inverse association was observed in studies conducted in Asia (*n* = 5, RR = 0.83, 95% CI 0.73–0.94) but not in studies from Europe (*n* = 4, RR = 1.02, 95% CI 0.91–1.16) or the USA (*n* = 7, RR = 0.95, 95% CI 0.77–1.16) (11). A similar geographical difference was also observed in another meta-analysis of 16 prospective cohort studies (12). No statistically significant association between the egg intake and risk of any stroke was observed in a meta-analysis of 17 prospective cohort studies (RR = 0.96, 95% CI 0.88–1.06 for highest vs. lowest category) (10). Regarding egg consumption and stroke mortality, three meta-analyses with 8 (8), 9 (11) and 9 (9) prospective cohort studies found an inverse association, while one meta-analysis with 6 prospective cohort studies found no association (12). For example, in the most recent meta-analysis, one egg/week increase in intake was associated with RR = 0.96 (95% CI 0.92–1.00) (9). Only one of the studies included in the meta-analysis was conducted in Europe, with RR = 1.07 (95% CI 0.69–1.66). The other meta-analyses did not explore differences by study location.

In summary, the strength of evidence is regarded as *Limited – suggestive* that when compared to low or no egg intake, moderate egg intake is associated with lower risk of stroke.

#### Heart failure

A meta-analysis of four prospective cohort studies found that compared to no egg consumption, ≥7 eggs/week was associated with an increased risk of heart failure (RR = 1.15, 95% CI 1.02–1.30), but the association was found in the only study conducted in the USA (RR = 1.32, 95% CI 1.11–1.58 for one egg/day higher intake), not in the three European studies (RR = 1.08, 95% CI 0.88–1.32) (12). The strength of evidence is regarded as *Limited – no conclusion*.

### Hypertension

One meta-analysis with three prospective non-European cohort studies investigated the association of egg intake with incident hypertension and found an inverse association (RR = 0.79, 95% CI 0.68–0.91 for highest vs. lowest category) (14). The strength of evidence is regarded as *Limited – no conclusion*.

### Type 2 diabetes

The most recent meta-analysis with 16 prospective cohort studies did not find a statistically significant association between egg intake and risk of T2D (RR = 1.07, 95% CI 0.99–1.15 for each one egg/day increase in intake) (15). When the analyses were stratified by study location, egg intake was associated with a higher risk in the US studies (*n* = 8, RR = 1.19, 95% CI 1.10–1.27), but no association was found in the studies conducted in Europe (*n* = 8, RR = 0.99, 95% CI 0.85–1.15) or in Asia (*n* = 2, RR = 0.82, 95% CI 0.62–1.09) (15). In summary, the strength of evidence is regarded as *Limited – suggestive* that egg intake is not associated with increased risk of T2D in European populations.

### Cancer

#### Cancer mortality

A meta-analysis with 13 prospective cohort studies found that higher egg intake was associated with higher risk of cancer death (RR = 1.20, 95% CI 1.04–1.39 for highest vs. lowest category) (9). When stratified by geographic location, the increased risk was found in the US studies (*n* = 4, RR = 1.33, 95% CI 1.11–1.60), but not in European studies (*n* = 3, RR = 1.10, 95% CI 0.84–1.41) or in studies from Asia (*n* = 6, RR = 1.15, 95% CI 0.92–1.44) (9).

#### Bladder cancer

A meta-analysis including four prospective cohort studies and nine case–control studies did not find an overall association between egg intake and risk of bladder cancer (RR = 1.11, 95% CI 0.90–1.35 for high vs. low intake) (16). When stratified by geographic location, egg intake was associated with higher risk in US studies (*n* = 5, RR = 1.40, 95% CI 1.05–1.86) but not in European studies (*n* = 4, RR = 1.01, 95% CI 0.79–1.30) or in Asian studies (*n* = 4, RR = 0.83, 95% CI 0.52–1.32). In two studies that had assessed the cooking method, higher intake of fried egg was associated with higher risk (RR = 2.04, 95% CI 1.41–2.95), whereas higher intake of boiled egg was not (RR = 1.25, 95% CI 0.82–1.91).

#### Gastrointestinal cancer

In a meta-analysis of seven prospective cohort studies and 37 case–control studies, egg intake of ≥3 eggs/week was associated with OR = 1.25 (95% CI 1.14–1.38) when compared to (presumably) no intake (17). Increased risk was found only in case–control studies but not in cohort studies. No associations were found in studies from Europe. In site-specific analyses, egg intake was associated mainly with risk of cancers of the stomach, colon, and colorectum.

#### Prostate cancer

A meta-analysis that included 10 prospective cohort studies found that egg intake was not associated with risk of total prostate cancer (RR = 1.00, 95% CI 0.88–1.14 for each five eggs/week higher intake) but was associated with higher risk of fatal prostate cancer (RR = 1.47, 95% CI 1.01–2.14) (18).

#### Brain cancer

In a meta-analysis of five case–control studies, higher egg intake was not associated with risk of brain cancer overall but was associated with a higher risk in the two studies with ≥200 cases (RR = 1.57, 95% CI 1.27–1.93 for highest vs. lowest category) (20).

#### Upper aero-digestive tract cancers

A meta-analysis found that egg intake was not associated with risk of upper aero-digestive tract cancers in analyses with four prospective cohort studies and two nested case–control studies (OR = 0.86, 95% CI 0.71–1.04 for highest vs. lowest intake category) but was associated with higher risk in case–control studies (OR = 1.42, 95% CI 1.19–1.68) (21). Increased risk was found with oropharyngeal cancer (OR = 1.88; 95% CI 1.61–2.20), laryngeal cancer (OR = 1.83, 95% CI 1.45–2.32), oral, pharyngeal or laryngeal cancer (OR = 1.37, 95% CI 1.12–1.67), and esophageal cancer (OR = 1.28, 95% CI 1.10–1.48). Increased risk was found only in hospital-based case–control studies, but not in population-based case–control studies.

#### Non-Hodgkin lymphoma

A meta-analysis including one prospective cohort study and six case–control studies did not find an association between egg intake and risk of non-Hodgkin lymphoma (RR = 1.15, 95% CI 0.87–1.51 for high vs. low intake) (19).

#### Breast cancer

A meta-analysis with 11 prospective cohort studies did not find an association between egg intake and risk of breast cancer (RR = 1.03, 95% CI 0.96–1.12 for each 50 g/day higher intake) (22).

#### Ovarian cancer

In a meta-analysis of nine prospective cohort studies, egg intake was not associated with risk of ovarian cancer (RR = 1.12, 95% CI 0.96–1.30) (23).

In summary, the strength of evidence is regarded as Limited – suggestive that egg intake is not associated with cancer risk in studies conducted in Europe. For the cancer outcomes for which geographic information is not available, the strength of evidence is regarded as *Limited – no conclusion*.

### Total mortality

A meta-analysis of seven prospective cohort studies did not find evidence for an association between egg intake and the risk of total mortality (RR = 1.11, 95% CI 0.97–1.26 for highest vs. lowest intake category) (8). A more recent meta-analysis with 24 prospective cohort studies came to the same conclusion (RR = 1.02, 95% CI 0.94–1.11 for highest vs. lowest category), although it found an association with increased risk in the studies conducted in the USA (*n* = 7, RR = 1.18, 95% CI 1.10–1.26), but not in Europe (*n* = 8, RR = 1.03, 95% CI 0.78–1.35) or in Asia (*n* = 9, RR = 0.89, 95% CI 0.80–0.99) (9). The strength of evidence is regarded as *Probable* that egg intake is not associated with risk of total mortality in European populations.

### Other outcomes

#### Metabolic syndrome

A meta-analysis including four prospective cohort studies and 14 cross-sectional studies found that higher egg intake was associated with a lower risk of metabolic syndrome (RR = 0.92, 95% CI 0.88–0.96) (24). However, the association was only found in the cross-sectional studies (RR = 0.91, 95% CI 0.88–0.95) not in the prospective studies (RR = 0.99, 95% CI 0.77–1.26). Only one of the studies in the meta-analysis was conducted in Europe. The strength of evidence is regarded as *Limited – no conclusion*.

## Mechanisms

Egg contains nutrients that may have beneficial or unfavorable effects on health. Several systematic reviews and meta-analyses of RCTs have investigated the effects of increased egg intake (commonly 1–4 eggs/day) on disease risk factors, mainly serum lipid profile, blood pressure, and inflammation markers. Most of the meta-analyses include a heterogenous group of studies with either healthy subjects or subjects with a history of disease, e.g. T2D, metabolic syndrome or coronary artery disease, or subjects with hypertension or hypercholesterolemia. However, many meta-analyses have reported the results stratified by the health status of the subjects. As the target group for the NNR2023 is the general population, the results from the studies with healthy subjects were considered relevant in this section.

The major potentially unhealthy nutrient in an egg is cholesterol. Also, choline has been suggested to have unfavorable health effects due to its conversion to TMAO. The potentially beneficial compounds in eggs include the bioactive compounds, such as protein-derived peptides, egg yolk lipids, the carotenoids lutein and zeaxanthin, and phosphatidylcholine and other phospholipids that may have beneficial effects on, e.g. inflammation, lipid oxidation, lipid and glucose metabolism, blood pressure, atherosclerosis progression, and cognitive performance (5, 55–62).

### Serum lipid profile

Egg intake, due to its high cholesterol content, may raise serum total and low-density lipoprotein (LDL) cholesterol (the ‘bad’ cholesterol) concentrations and therefore increase the risk of CVD. The apolipoprotein-B-containing lipoproteins, especially LDL, have a major causal role in the development of atherosclerotic CVD (63). High egg intake may also increase the serum high-density lipoprotein (HDL) cholesterol (the ‘good’ cholesterol) concentrations, although the role of increased HDL cholesterol concentrations in atherosclerotic CVD is still unclear (63). One medium-sized egg (55 g) contains about 200 mg of cholesterol, which is 2/3 of the 300 mg/day that was recommended as the maximum amount of dietary cholesterol in several guidelines in the past. However, such specific limits have been removed from many of the recent guidelines.

A meta-analysis of 17 RCTs with healthy subjects found a higher LDL/HDL cholesterol ratio and higher LDL cholesterol concentrations with increased egg intake, without evidence for significant heterogeneity between the studies (50). For example, the mean LDL cholesterol concentration was 0.21 mmol/L (95% CI 0.12–0.31 mmol/L) higher in the egg group vs. the control group. Another meta-analysis found that the intake of >1 egg/day increased serum total cholesterol (48 studies with healthy subjects, mean difference 0.26 mmol/L, 95% CI 0.20–0.31 mmol/L), LDL cholesterol (40 studies in healthy subjects, mean difference 0.20 mmol/L, 95% CI 0.15–0.25 mmol/L), and HDL cholesterol (44 studies in healthy subjects, mean difference 0.04 mmol/L, 95% CI 0.02–0.06 mmol/L) concentrations and the total/HDL cholesterol ratio (14 studies in healthy subjects), but there was evidence for significant heterogeneity (51).

The impact of egg intake on the serum LDL cholesterol concentration may be more pronounced among those who are so called hyperresponders to dietary cholesterol. A Finnish RCT reported in 1998 that adding two egg yolks to a diet for 4 weeks had a greater impact on the serum LDL cholesterol concentration among subjects with the apolipoprotein-E4/4 genotype than among subjects with the E3/4 or E3/3 genotypes (64). However, there are no meta-analyses on this topic as there is still very limited research on the impact of the apolipoprotein-E genotype (and other potential genetic factors) on serum lipid responses due to high egg intake.

### Trimethylamine-N-oxide

Egg is a major source of choline in the diet, with about 140 mg of choline in one egg. This accounts for about 35% of the adequate intake level of 400 mg/day for adults set by the European Food Safety Authority Panel (65). Although choline is an essential nutrient that is needed as a precursor for the neurotransmitter acetylcholine and for the membrane constituent phosphatidylcholine and is required for normal liver and brain function, it is also metabolized into trimethylamine by gut microbiota and then further in the liver to TMAO, a purported risk factor for CVD (3). However, the data on the impact of egg intake on the TMAO production are controversial, with substantial interindividual variation in the response (66). A major source of variability may be the differences in gut microbiota because not all strains of bacteria are capable of converting choline to trimethylamine (67).

### Blood pressure

A meta-analysis of 15 RCTs (8 among healthy subjects) found no significant effect of increased egg consumption on systolic or diastolic blood pressure overall or in the studies with healthy subjects (49).

### Inflammation

A meta-analysis of nine RCTs (five with healthy subjects) did not find an effect of increased egg intake on inflammation markers in all studies or in studies with healthy subjects (48).

## Food-based dietary guidelines

Although RCTs suggest that high egg intake (>1 egg/day) may increase serum LDL cholesterol concentrations, prospective cohort studies have found that moderate egg intake (up to 1 egg/day) may be associated with lower risk of certain CVD outcomes, such as non-fatal CVD and CHD and stroke, although this may be limited to studies conducted in Asia. A clear biological mechanism by which moderate egg consumption might lower the risk of CVD is lacking. In contrast, intakes of ≥1 egg/week have been associated with an increased risk of overall CVD and heart failure. The latter finding is difficult to interpret considering the lack of biological mechanisms behind this association. Furthermore, the increased risk of CVD and heart failure was mainly observed in the US studies, limiting the generalizability of the findings and further suggests that residual confounding or other biases may explain the observed associations.

Regarding the risk of T2D, the lack of an overall association in the studies conducted in Europe or in Asia and the association with higher risk in the studies from the US may reflect different egg consumption habits between populations and residual cofounding from correlated food intakes and other risk factors for T2D in the US studies.

The totality of evidence from observational studies on egg consumption and cancer risk provides limited support for a role of egg consumption as a risk factor for major cancers, although a modestly elevated risk of certain cancers associated with egg consumption cannot be ruled out. However, the evidence for a higher risk of certain cancers comes mainly from retrospective case–control studies that have a substantial risk of selection and recall bias in nutrition research and are a source of significant heterogeneity in the case of most cancers (Table 1). Considering the observational design of the available studies, any observed weak association may be driven by residual confounding from other risk factors for cancers, such as other dietary factors, obesity, physical inactivity, and smoking. Finally, as with CVD and T2D, the increased risk has been mainly observed in the studies conducted in the USA.

### Data gaps for future research

As there are no RCTs about the effects of egg intake on the incidence of diseases, the evidence for the relationship of egg intake with disease risk is based on the findings from observational studies, which are susceptible to residual confounding and reverse causation bias. For example, results of a prospective study of 409,885 adults in nine European countries (the European Prospective Investigation Into Cancer and Nutrition study) showed that egg consumption was associated with lower risk of ischemic heart disease, but the association was attenuated after removing the first 4 years of follow-up, suggesting that reverse causation bias may have influenced the results in the overall analysis (68). Similarly, in observational studies, higher egg intake has been associated with higher CVD risk in subjects with T2D (10, 12), but RCTs among subjects with prediabetes or T2D have found little evidence for adverse effects on CVD risk factors with increased egg intake in studies lasting up to 12 months (47, 69).

Because of the financial and practical limitations, for most dietary factors, there will never be RCTs that would investigate the effects on incidence of diseases. Also, most RCTs have evaluated the impact of increased egg intake only on few ‘traditional’ disease risk markers, such as serum lipid profile, blood pressure, and inflammation, which may not give a complete picture of the health effects of egg intake. However, short-term RCTs with a comprehensive panel of examinations and measurements, including more advanced measurements, such as wide-scale ‘-omics’ techniques, would give a detailed image of the physiological and metabolic effects of high egg intake. Currently, there are no such studies for most dietary factors, including eggs. Furthermore, there is no research on whether high egg intake could influence gut microbiota in humans, although animal models have suggested beneficial effects with certain bioactive compounds that are rich in eggs (70, 71). On the other hand, there is also very little research data whether the composition of the gut microbiota has an influence on the physiological effects of egg intake, as has been suggested in the case of TMAO production from choline (66).

Another important topic with limited research data is the overall health effects of high egg intake among the hyperresponders to dietary cholesterol. Although high egg yolk intake increased serum LDL cholesterol concentrations most among those with the apolipoprotein-E4 genotype in a Finnish RCT (64), a Finnish observational study found that higher egg intake did not associate with carotid atherosclerosis or risk of CHD or stroke even among the participants with the apolipoprotein-E4 genotype (72, 73). There are little similar research data from experimental studies for the impact of the apolipoprotein-E genotype (or other genetic factors) on other disease risk factors besides serum cholesterol concentrations.

Because egg is a rich source of lutein and zeaxanthin, two carotenoids that largely maintain macular pigment function, there has been interest in the impact of egg intake for the prevention of age-related macular degeneration, which leads to loss of vision. A recent meta-analysis of five RCTs concluded that egg intake may reduce the progression of age-related macular degeneration (74). However, some of the studies used lutein-enriched eggs and when the analyses were stratified by the intervention type (normal eggs or lutein-enriched eggs), the results did not reach statistical significance anymore. More evidence from RCTs and observational studies is needed before any conclusions can be drawn about the impact of egg intake on eye health.

Finally, several observational studies have suggested that moderate egg intake may have a neutral or an inverse association with the risk of cognitive decline or mortality from neurodegenerative diseases, including Alzheimer’s disease (75–80). One possible explanation is the high choline and lutein content in eggs (81, 82). There are no systematic reviews or meta-analyses of this topic, so this outcome was not included in this scoping review. Currently, there is no evidence from RCTs whether increased egg intake could have an effect on cognitive decline, although such studies would be warranted based on the findings in observational studies, and the fact that the number of people with Alzheimer’s disease and other dementias is increasing around the world (83).

### Limitations

A major limitation in the meta-analyses of observational studies is that they seldom consider the replacement food. In RCTs with egg intake, the control group has most often consumed either no eggs, egg whites, lean animal protein, egg substitutes, or oatmeal. In observational studies, if higher egg intake is associated with a disease risk, the question to ask is ‘compared to what?’ Compared to all other foods in the diet or just to certain specific food(s)? Such substitution analyses are unfortunately rarely done in the observational studies, although the comparison food(s) may have a significant impact on the observed associations of egg intake with disease risk. This is nicely illustrated in the editorial (84) to the recent meta-analysis of egg intake and CVD risk (13). This may also at least partly explain the significant heterogeneity with most disease outcomes (as shown in Table 1) and the geographical differences in the risk estimates.

Another limitation is that most observational studies have investigated the associations with disease risk with up to one egg/day because only a small proportion of the study populations commonly consume higher amounts. Therefore, there are little data for the associations between long-term intake of more than one egg/day and risk of diseases. Experimental studies have commonly used higher amounts of eggs (1–3 eggs/day), but in contrast, the studies usually last only for a few weeks or months, again making it difficult to draw conclusions of the long-term health effects of high egg intake. Finally, in the current evidence base, there are very little research data on the health effects of egg intake on children or adolescents or on pregnant or lactating women.
